# High-resolution Imaging of Myeloperoxidase Activity Sensors in Human Cerebrovascular Disease

**DOI:** 10.1038/s41598-018-25804-y

**Published:** 2018-05-16

**Authors:** Youssef Z. Wadghiri, Dung Minh Hoang, Anita Leporati, Matthew J. Gounis, Aurora Rodríguez-Rodríguez, Mary L. Mazzanti, John P. Weaver, Ajay K. Wakhloo, Peter Caravan, Alexei A. Bogdanov

**Affiliations:** 1 New York University School of Medicine, Department of Radiology, Centre for Advanced Imaging Innovation & Research (CAI2R) and Bernard & Irene Schwartz Centre for Biomedical Imaging, New York, NY USA; 2 Department of Radiology, University of Massachusetts Medical School, Worcester, MA USA; 3 A. Martinos’ Centre for Biomedical Imaging, Massachusetts General Hospital, Charlestown, MA USA; 4 Department of Neurosurgery, University of Massachusetts Medical School, Worcester, MA USA

## Abstract

Progress in clinical development of magnetic resonance imaging (MRI) substrate-sensors of enzymatic activity has been slow partly due to the lack of human efficacy data. We report here a strategy that may serve as a shortcut from bench to bedside. We tested ultra high-resolution 7T MRI (µMRI) of human surgical histology sections in a 3-year IRB approved, HIPAA compliant study of surgically clipped brain aneurysms. µMRI was used for assessing the efficacy of MRI substrate-sensors that detect myeloperoxidase activity in inflammation. The efficacy of Gd-5HT-DOTAGA, a novel myeloperoxidase (MPO) imaging agent synthesized by using a highly stable gadolinium (III) chelate was tested both in tissue-like phantoms and in human samples. After treating histology sections with paramagnetic MPO substrate-sensors we observed relaxation time shortening and MPO activity-dependent MR signal enhancement. An increase of normalized MR signal generated by ultra-short echo time MR sequences was corroborated by MPO activity visualization by using a fluorescent MPO substrate. The results of µMRI of MPO activity associated with aneurysmal pathology and immunohistochemistry demonstrated active involvement of neutrophils and neutrophil NETs as a result of pro-inflammatory signalling in the vascular wall and in the perivascular space of brain aneurysms.

## Introduction

**Figure 1:**
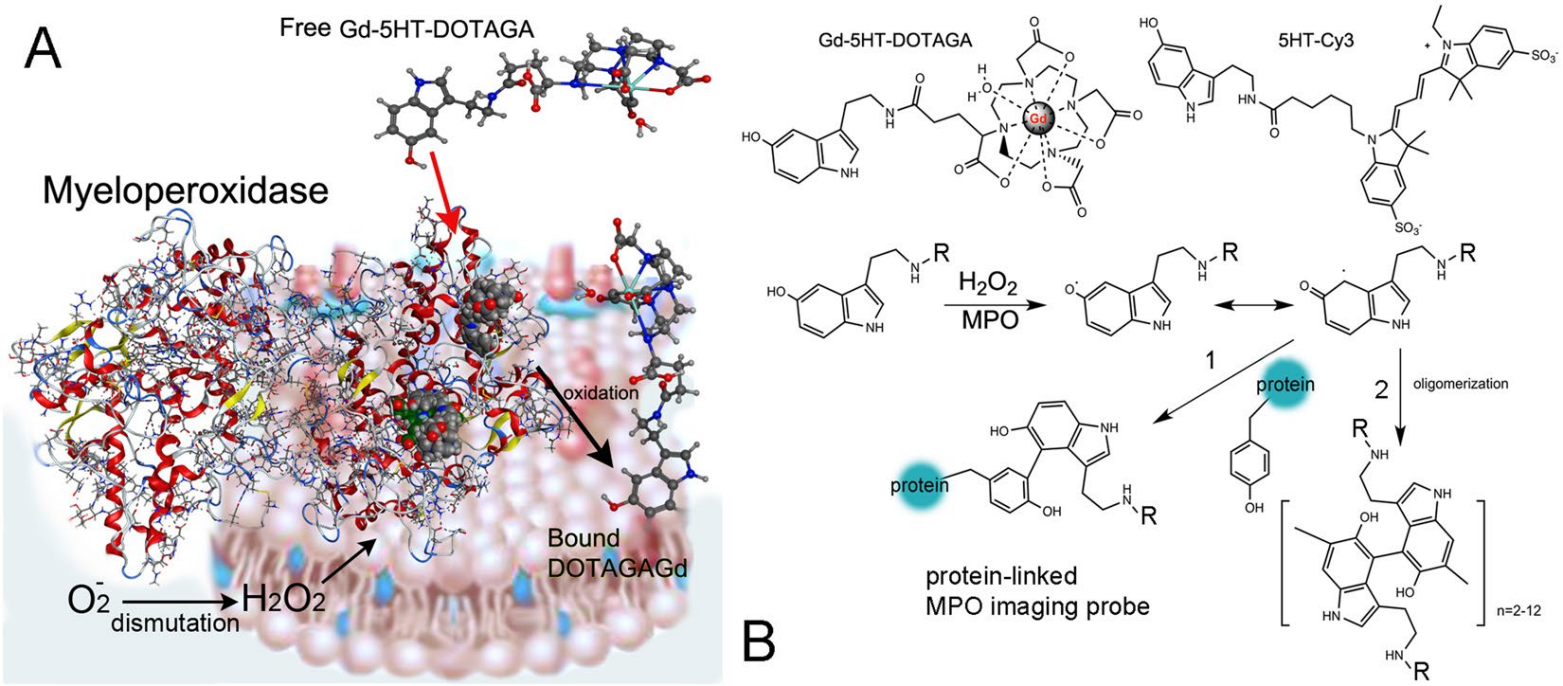
MPO-mediated reactions of imaging probes resulting in imaging signal. (**A**) Membrane-associated myeloperoxidase (MPO) dimer converts soluble paramagnetic substrates into the immobilized products which increase paramagnetic MR signal. Hydrogen peroxide is generated in the blood vessel wall as a result of superoxide anion dismutation. MPO is using hydrogen peroxide and Gd-5HT-DOTAGA (shown as an example) to generate oxidized products at protoporphyrin IX MPO catalytic sites. These products bind to nearby proteins. (**B**) Chemical formulas of Gd-5HT-DOTAGA and 5HT-Cy3. The simplified reaction mechanism between the hydroxytryptamide probes (R = Gd-DOTAGA or Cy3) and the oxidized form of MPO is shown below. The intermediates (free radicals) react with the proteins at tyrosine residues (pathway 1) or undergo dimerization/oligomerization (pathway 2)^19,49^.

Magnetic resonance imaging (MRI) provides 3-D structural information about tissue volume as well as detailed physiological and anatomical information for thick tissue sections. Ultra-high-resolution MRI using small field-of-view acquisitions, i.e. micro-MRI (µMRI), can be used to obtain microscopic images within whole organs (e.g. MRI biopsy) reflecting differences in water relaxation times in various tissue components^1^. Such µMRI techniques have been used to study the developmental biology of mice^2,3^, reveal details of brain structure^4,5^, and show anatomical and functional changes of skeletal/connective tissue systems^6,7^. Moreover, development of specially designed radiofrequency coils has further allowed µMRI of sub-millimetre thick microscopic tissue sections^8,9^. Both non-specific^10^ and pathology-specific MR contrast agents^5^ have been evaluated for their ability to produce images with highly delineation of target areas and their compatibility with standard histology markers. However, the utility of µMRI as a surrogate for high-resolution contrast-assisted *in vivo* MR imaging has not been fully explored. Using animal models of human disease, we and others previously demonstrated that paramagnetic substrate-sensors of neutrophil myeloperoxidase (MPO) activity^11,12^ are highly useful in imaging inflammation-linked pathologies of the cardiovascular system^13–15^ and brain^16,17^. Generation of MPO-specific MR contrast is brought about via the enzymatic oxidation of MPO-reducing hydroxytryptamides. These paramagnetic hydroxytryptamides are small molecules, which undergo various oxidative transformations ultimately leading to the formation of dimers, oligomers and protein-bound forms^12,18^. Covalent binding to proteins results in the retention of paramagnetic chelated metals on cell surfaces and components of the extracellular matrix^19,20^, Fig. 1A. Here we report the utility of µMRI for detecting MPO activity in inflammatory lesions within the vascular wall of excised human brain aneurysms (hBA) and arteriovenous malformations (AVM). By combining µMRI with fluorescent probe-mediated imaging of MPO activity (Fig. 1B) and immunohistochemical corroboration we set forth to find out: (1) whether µMRI of enzyme- dependent vascular wall enhancement is feasible in the very small volumes of histology sections and; (2) whether such MR signal enhancement correlates with the distribution of MPO activity in the tissue.

## Results

### Histology of human tissue samples

**Figure 2:**
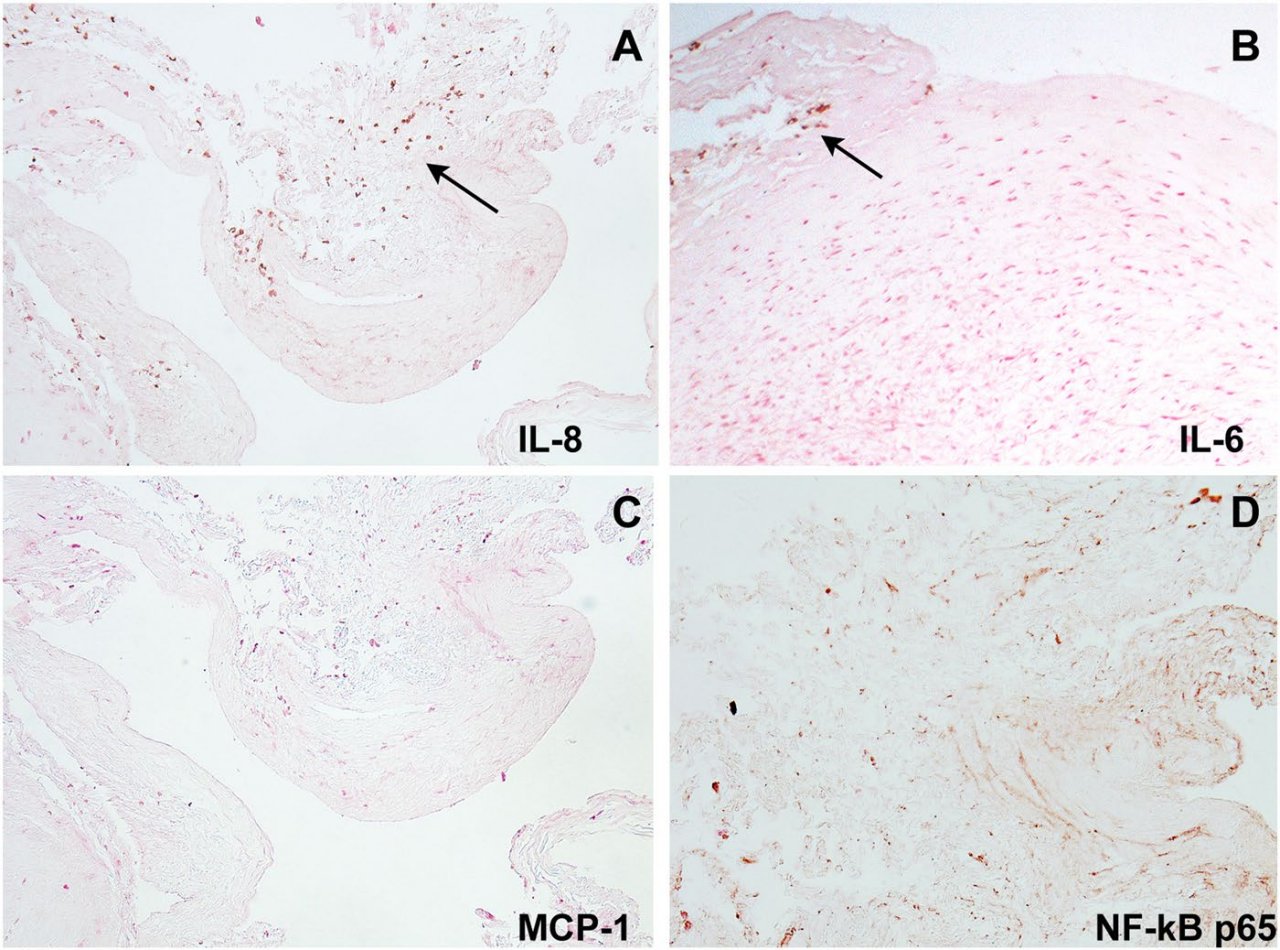
Immunohistochemistry of pro-inflammatory marker expression in a representative unruptured hBA. Immunoperoxidase detection of the following antibodies: (**A**) anti-IL8; (**B**) anti-IL6; (**C**) anti-MCP-1; (**D**) anti-NF-kB phospho p65. The sections were counterstained with H & E. Arrows point to cytokine- positive cells in panels A and B. Magnification – 20x.

The initial immunohistochemistry of unruptured hBA samples obtained as a result of surgical clipping revealed multiple IL-8 (CXCL8) cytokine positive cells infiltrating the vascular wall both along the adventitial and luminal sides of hBA (Fig. 2A,C). Ineterleukin-6-positive cells were present as sparse infiltrates detectable only on the adventitial side of the vessels (Fig. 2B) while MCP-1 (CCL2) antigen was not detectable in any of the samples we tested. Smooth muscle cells in hyperplastic thickened areas of aneurysmal wall showed a strong activation of NF-kB with nuclei of cells binding an antibody against phosopho-p65 in all unruptured aneurysmal samples we processed (Fig. 2C).

**Figure 3:**
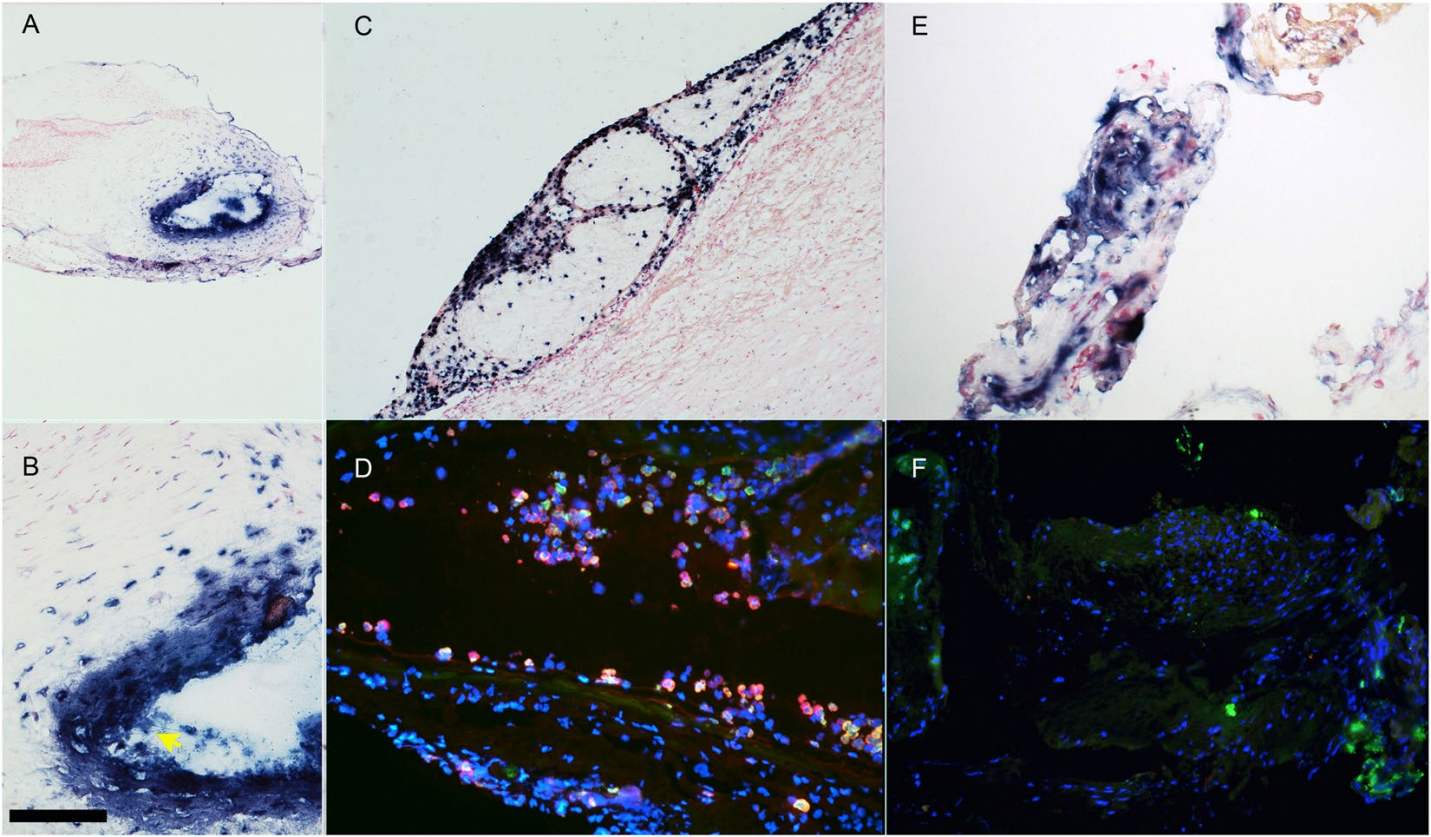
Immunohistochemistry and immunofluorescence of clipped human unruptured hBA (thin frozen sections). (**A**) (magnification – 10x), (**B**) (magnification – 40x) – hBA, yellow arrow points to MPO-positive NETs in the lumen, bar = 100 µm, anti-MPO staining with anti-human MPO monoclonal antibody (alkaline phosphatase-labelled secondary anti-mouse mAb); (**C**) hBA (magnification – 20x), anti- MPO staining. (**D**) hBA dual staining of infiltrated periluminal neutrophils with anti-MPO (red fluorescence) and anti-elastase (green fluorescence) antibodies, 20x; (**E**) hBA, anti-MPO staining, 20x; (**F**) hBA anti-elastase (green fluorescence) staining. In panels A, B, C, and E the nuclei are counterstained with nuclear fast red. In D and F nuclei are stained with DAPI (blue).

**Figure 4:**
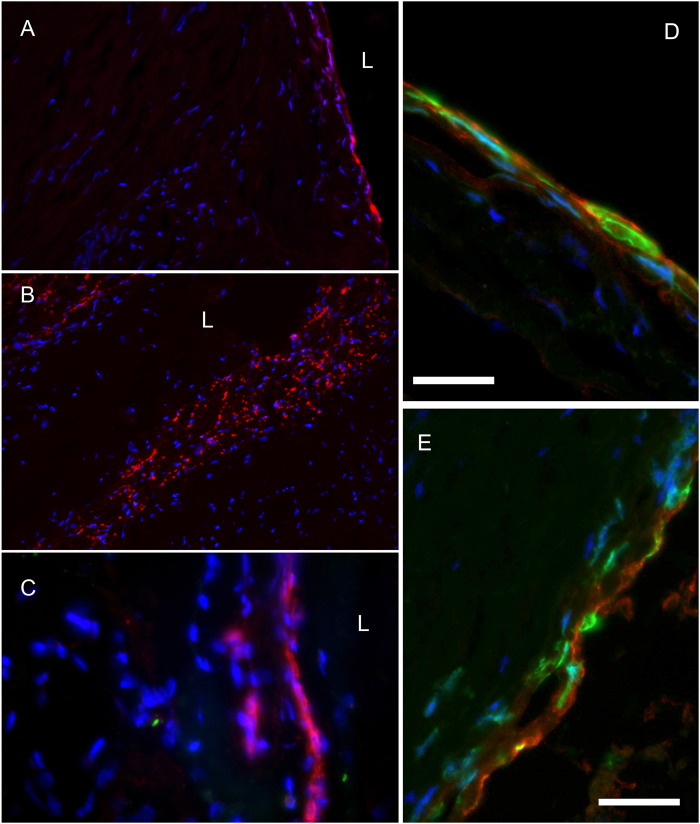
Fluorescence microscopy of MPO activity. The images show 5HT-Cy3 staining of MPO – positive cells. 5HT-Cy3 (red fluorescence) detects the presence of active MPO enzyme. (**A**) The infiltration of MPO+ neutrophils from the vascular lumen of human unruptured hBA; (**B**) MPO in cells infiltrated in AVM (magnification – 10x); (**C**) AVM sample showing intracellular and extracellular MPO at high magnification (40x), L- vascular lumen; (**D**) high-magnification image of infiltrating neutrophils in unruptured hBA (see panel A) by using MPO and anti-calprotectin (S100A8/A9 complex) detection (63x); (**E**) - high-magnification image of infiltrating neutrophils in AVM (see panel C) by using MPO and calprotectin detection. Red- 5HT-Cy3, green - anti-calprotectin antibody, blue - DAPI. Bars (**D,E**) = 25 µm.

As determined by our interdisciplinary cerebrovascular interventional imaging team, immunohistochemistry and immunofluorescent detection of specific neutrophil enzymatic markers in tissue sections demonstrated that regardless of hBA location, all unruptured hBA contained neutrophil-specific antigens, i.e. myeloperoxidase (Fig. 3A–D) and elastase (Fig. 3D,F). Both enzymes could potentially be used as tracking markers of neutrophils. We observed the presence of intracellular and extracellular neutrophil MPO antigen in the majority of samples of clipped brain aneurysms. Neutrophils were localized primarily around the lumen after infiltrating into the vascular wall (Fig. 3B–D), as well as within the walls of the adventitial neovessels^21,22^. Intracellular neutrophil elastase was also present and could be identified by using a single or dual staining (alongside with anti-MPO antibody, Fig. 3D,F). The vessel wall-infiltrated neutrophils showed more prominent anti-elastase staining than more peripheral MPO-positive neutrophils attached to the surface of the aneurysms (Fig. 3D). In the subluminal regions of the vessel walls we observed the formation of perivascular wall haemorrhages and neutrophil extracellular traps (NETs) suggesting terminal neutrophil activation in the areas of vascular pathology and microthrombosis (Fig. 3B, arrow). Furthermore, by using a fluorescent MPO substrate 5HT-Cy3 (5-hydroxytryptamide of the cyanine dye Cy3, a red fluorophore) we were able to perform visualization of MPO activity in surgical samples. The assessment of MPO activity as opposed to the presence of protein antigen alone was essential for determining whether it would be feasible to further process the samples for µMRI because MR signal depended on the presence of active MPO enzyme. Activation of 5HT-Cy3 by MPO results in production of reactive species that link to nearby molecules of which a substantial fraction are proteins of extracellular matrix and fibrin of thrombi. By incubating frozen sections with 5HT-Cy3 in the presence of hydrogen peroxide we observed MPO activity as bright red fluorescent signal associated with the cells infiltrating human AVM and hBA samples (Fig. 4A–C). It should be noted that in the majority of processed human samples, MPO activity was positively identified in polymorphonuclear cells as well as extracellular deposits (Fig. 4C) suggesting the stability of extracellular enzyme resulting in preservation of enzyme activity. These cells had irregular shaped nuclei and were present in both unruptured hBA and AVM surgical samples. The cells stained positively not only for MPO activity with 5HT-Cy3 probe, but also expressed S100 calcium-binding proteins characteristic of granulocytes and some other leukocytes (Fig. 4D,E).

### Testing MPO substrates *in vitro*

To achieve MPO-specific µMRI imaging in histology sections we used two types of substrate-sensors: (1) Gd-bis-5HT-DTPA that was previously used by multiple research groups for imaging of MPO activity *in vitro* and *in vivo*^14–16^ (2) novel MR imaging sensor Gd-5HT-DOTAGA. All tested MPO imaging sensors function as electron donors, which is an essential property required to reduce oxidized MPO to a catalytically active state. Initially we tested both substrates in parallel to investigate whether the substitution of the acyclic DTPA chelate for a macrocyclic DOTAGA results in the expected increase of stability and thus resistance to trans-metallation, i.e. the release of paramagnetic Gd(III) from the chelate in the presence of an excess of competing biologically relevant ions such as Zn(II) and phosphate. As we anticipated, in transmetallation test Gd-5HT-DOTAGA showed no statistically significant decrease of relaxation rate R1 (p > 0.05). A decrease in R1 would signify a conversion into insoluble form and disappearance of Gd(III) from the solvating water environment. In contrast, Gd-bis-5-HT-DTPA showed a time-dependent decrease of the initial R1 values consistent with the lower stability of this MPO substrate-sensor (i.e. greater propensity for trans-metallation, see Supplementary Figure S1A).

We further tested whether the MPO-mediated catalysis reaction leads to a conversion of both macrocyclic and acyclic linear Gd(III) chelating substrates into products with higher Gd(III) molar relaxivity, which underlie the enhancement of MR signal in T1-weighted images. The effects of MPO reaction on the relaxivity of the initial substrates were analysed at three different field strengths. At low magnetic field strength (0.47T, 20 MHz) we observed the expected increase of molar longitudinal relaxivities of Gd(III) (r1, Figure S2). Both substrates (Gd-bis-5HT-DTPA and Gd-5HT-DOTAGA) resulted in a similar, approximately 1.5–1.7 fold increase of molar transverse relaxivity after MPO and hydrogen peroxide were added to reaction mixtures (Figure S1B). The increase was even more pronounced in the case of Gd-5HT-DOTAGA/MPO reactions run in the presence of human serum albumin (2.5-fold increase of longitudinal relaxivity) while Gd-bis-5HT-DTPA showed a lower relative increase. An increase of r1 due to MPO-catalysed reaction of Gd-5HT-DOTAGA was also seen at the higher clinical field strength of 1.4T. Unlike the measurements performed at lower magnetic field strengths (i.e. 0.47 and 1.5T), the measurements performed at 7T did not show any detectable Gd(III) relaxivity change when MPO reaction products were compared to the initial substrate (Figure S1B). Measurements of T2 and T2* of Gd-5HT-DOTAGA products of the reaction with MPO pointed to a strong increase of transverse relaxation effects, i.e. a sharp increase of r2/r1 ratio when compared to low field/lower resonance frequency environments.

### Micro-MRI and histology corroboration

**Figure 5:**
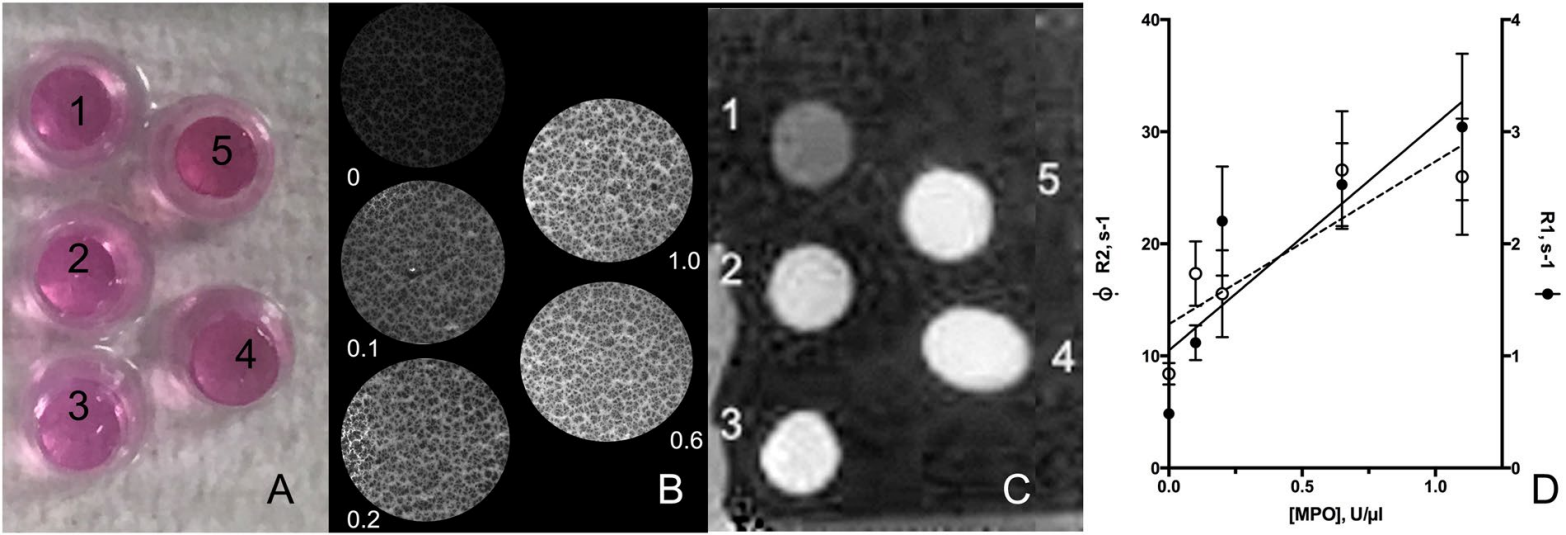
Fluorescence and 7T µMRI imaging of a tissue-like phantom. Phantom consisted of 5 wells containing thick (100 µm) frozen sections of tissue-like material containing various specific enzymatic activity of MPO (well 1 = 0, well 2 = 0.1, well 3 = 0.2, well 4 = 0.6 and well 5 = 1.0 U MPO/µl). MPO was dissolved and embedded in agarose/acrylamide/Matrigel mixture. (**A**) Phantom appearance, the colour is due to phenol red in the Matrigel samples 1–5 immersed in OCT medium; (**B**) fluorescence microscopy of thin sections of the phantom in A treated with 0.1 mM 5HT-Cy3/1 mM H_2_O_2_ after removing of unreacted 5HT-Cy3; (**C**) µMR imaging of the phantom after incubating 0.1 mm-thick sections in the presence and 0.5 mM bis-5HT-Gd-DTPA/1 mM H_2_O_2_ (UTE pulse sequence acquisition, with image inversion); (**D**) Relaxation rates R1 and R2 (measured by using µMRI of thick sections) as a function of MPO concentration in the phantom components. Data shown as mean ± SD (n = 3).

The verification of MPO activity in human tissue sections by a fluorescent 5HT-Cy3 substrate (Fig. 4) and subsequent testing of paramagnetic tryptamides (Figure S1) suggested the feasibility of detecting MPO paramagnetic substrate-sensors in human tissue using µMRI histology. We first validated the sensitivity of our µMRI system by performing parallel fluorescent and MR imaging of specially designed tissue-like phantoms. These phantoms were designed to obtain a model tissue containing MPO within thick and thin histological sections. Phantoms were made by moulding standard MPO solutions with known specific activity and concentration into separate cylindrically shaped gels by adding a polyacrylamide-stabilized Matrigel/agarose matrix. This approach (1) enabled imaging of samples containing various concentrations of MPO simultaneously, (2) allowed diffusion of MPO substrate-sensors into the matrix of phantom sections during co-incubations; (3) allowed testing of various MPO substrate-sensors simultaneously and (4) allowed measurement of water relaxation times after subsequent retention of MPO reaction products in the matrix of phantom sections. The range of MPO concentrations tested was chosen to emulate the range previously measured by our group in surgical samples of human aneurysmal tissue, i.e. approximately 200 U of MPO activity per mg-tissue in MPO-positive aneurysms^21^. Measurements of fluorescence intensity demonstrated that the average fluorescence intensity of MPO sample sections increased in order of increasing concentrations of MPO in the matrix (Fig. 5A,B); r1 and r2 molar relaxivities along with mean MR signal intensities were measured following 7T µMR acquisitions of embedded MPO samples. Results showed a near-linear correlation of MR R1 and R2 relaxation rate values (r^2^ = 0.76–0.78, p = 0.04–0.05) with increasing MPO concentrations (0.1–1.0 U MPO/µl) in section volumes of the phantom (Fig. 5C,D) and demonstrated the sensitivity of our µMRI imaging system.

**Figure 6:**
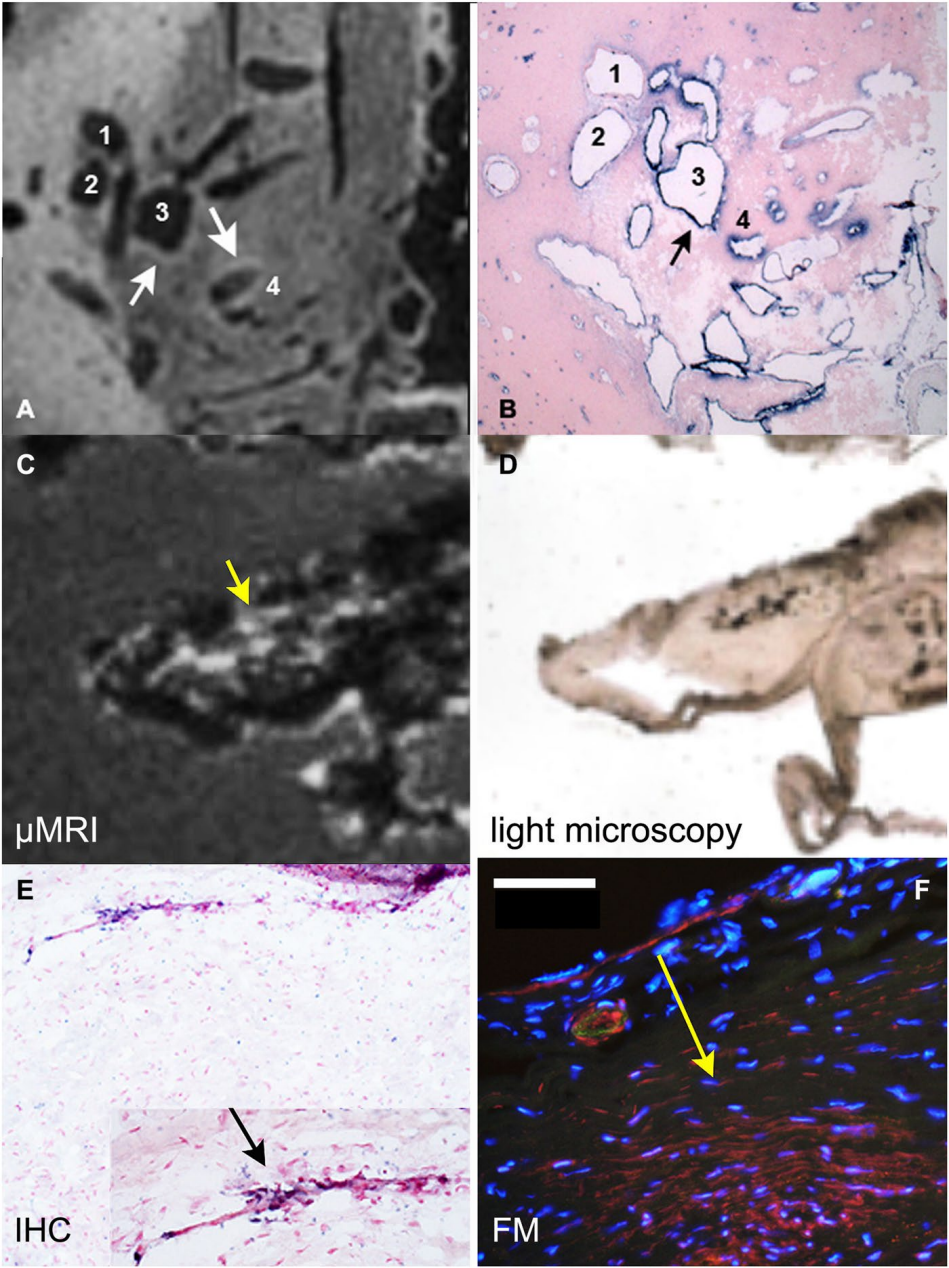
µMR Imaging and correlative immunohistochemistry of human arteriovenous malformation and unruptured human aneurysm. (**A**) µMRI image of a thick section (80 µm) of the arteriovenous malformation (AVM) showing moderate to strong T1-weighted signal detected over the circumference of the structures 3 and 4; (**B**) matching section showing immunohistochemical (IHC) detection of MPO (blue); (**C**) µMRI image of a left middle cerebral artery unruptured human aneurysm tissue sample; (**D**) a light microscopy image of thick section shown in panel C; (**E**) an anti-MPO IHC image (thin section). MPO positive cells (blue, 20x magnification) are pointed to by an arrow (inset). (**F**) Red fluorescence of MPO+ cells and extracellular MPO. Anti-MPO staining was performed by treating the tissue sequentially with anti-MPO mAb (anti-human mouse monoclonal antibody (1:200, AbCam), followed by a secondary mAb-alkaline phosphatase conjugate (1:500, Roche.). Yellow arrow is pointing to the area matched to the inset of panel (E, black arrow). This area is positive by MPO immunofluorescence (**E**) and MPO activity (**F**). Bar = 50 µm. In B and C the counterstaining of nuclei was performed by using Nuclear Fast Red (Vector labs) and DAPI was used for image F. MPO activity was revealed by incubating the section in the presence of 0.1 mM 5HT-Cy3/1 mM H_2_O_2_ for 30 min.

Next, by using the same µMRI acquisition set up we set out to investigate the utility and sensitivity of µMRI for the detection of inflammation in human brain tissue as evidenced by the presence of MPO activity and neutrophil infiltration in human brain tissue. Initially we performed imaging of sections prepared from a sample of human brain arteriovenous malformation (AVM) because it provided a larger sample volume and thus better coil-filling factor, which enables faster image acquisition and optimization (Fig. 6). Despite the endogenous bright contrast due to white matter present in the field of view, the imaging of AVM showed the presence of enhancement around the irregular vascular luminae (shown by arrows, Fig. 6A). This pattern of T1w-SE sequence generated signal enhancement (Fig. 6A), which correlated with the areas that were strongly reactive with anti-MPO antibody on histology sections (Fig. 6B).

**Figure 7:**
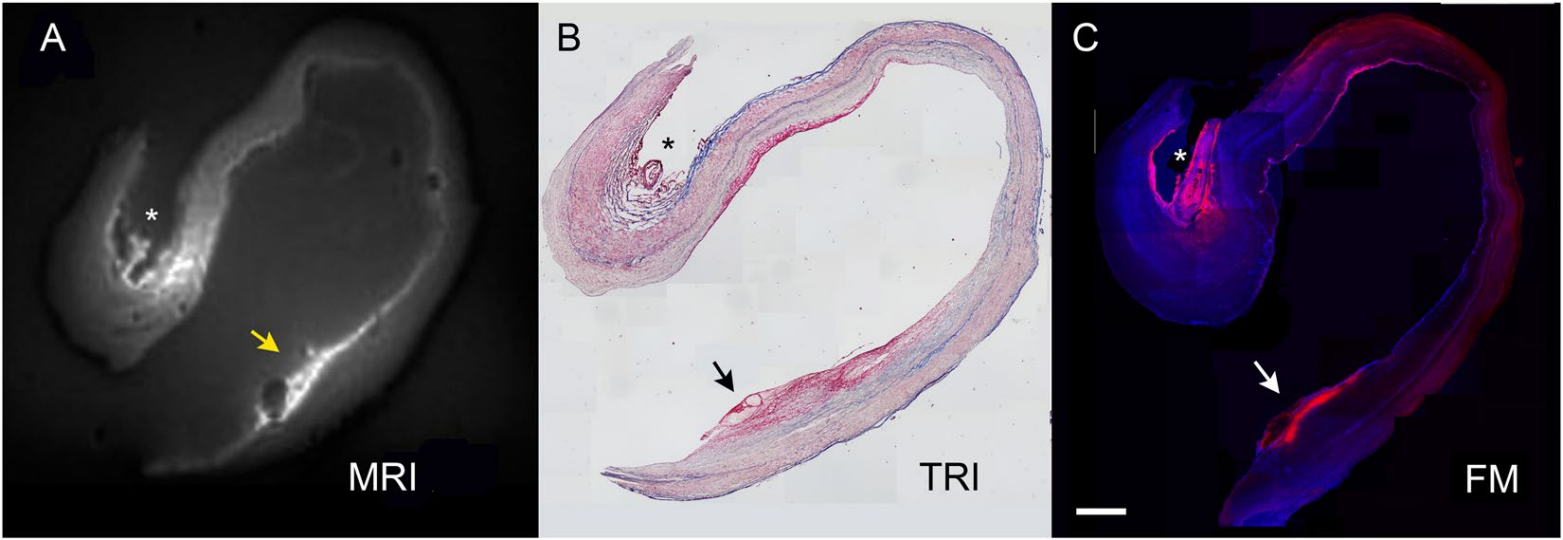
Comparison of µMRI of large unruptured hBA with the results of histology and fluorescence microscopy. (**A**) MRI (UTE pulse sequence) of a thick section of hBA treated with Gd-bis-5HT-DTPA and imaged by using µMRI coil; (**B**) trichrome stain of a consecutive thin section, magnification -10x; C- fluorescence microscopy of a thick section stained by using fluorescent MPO reducing substrate 5HT-Cy3, magnification -4x. Scale bar = 1 mm. Arrowheads point to the area of thrombus containing infiltrated neutrophils and MPO. Asterisks indicate the location of adventitial vessel (evidence of adventitial angiogenesis), containing MPO-positive cells.

**Figure 8:**
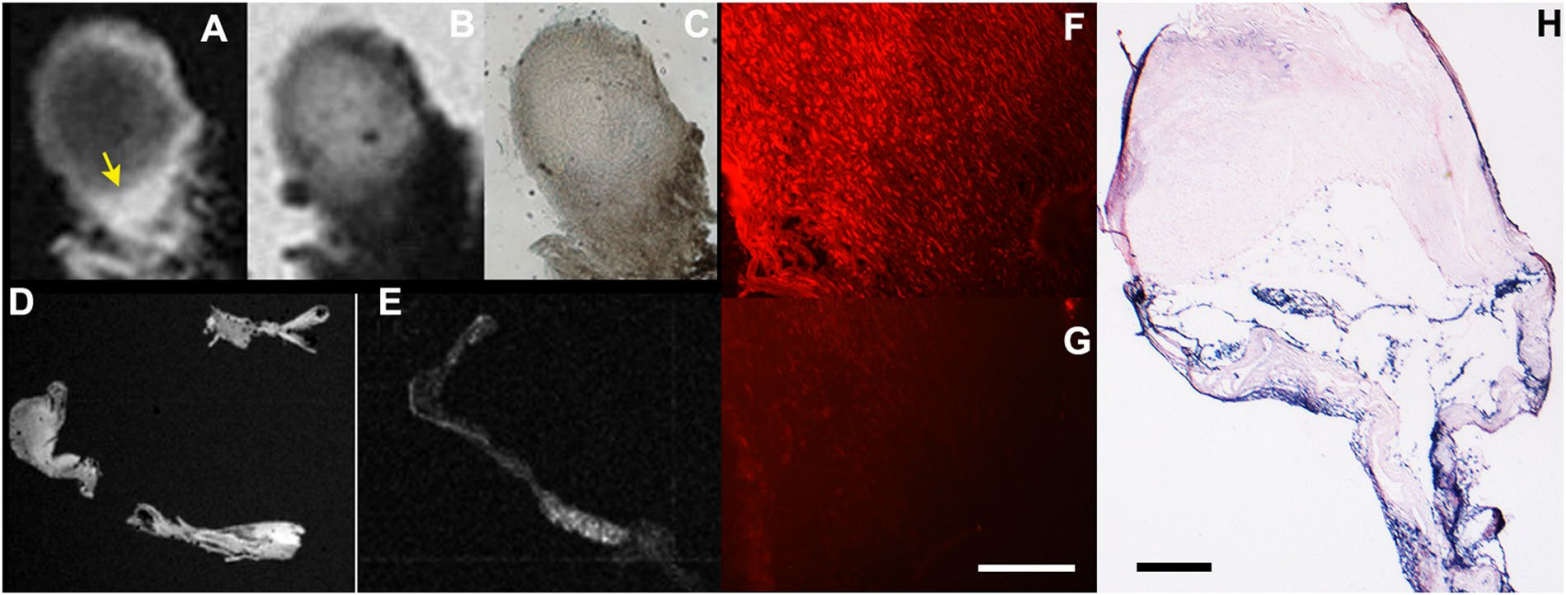
µMR Imaging and histology of ruptured hBA: (**A**) a T1w µMRI image of a thick section (80 µm) of ruptured hBA showing an area of strong enhancement in the dome of the aneurysm marked by an arrow; (**B**) T2w image of the same area; (**C**) light microscopy; (**D**) T1w µMRI image of vascular wall fragments in the same sample; (**E**) T1w µMRI of a section consecutive to (**D**) incubated with MPO paramagnetic substrate and 0.5 mM MPO inhibitor 4-aminobenzoichydrazide (4-ABH); (**F**) fluorescence microscopy image of a section corresponding to area on image (**A**) shown by the arrow after treating with 0.1 mM 5HT-Cy3/1 mM H_2_O_2_ to reveal MPO activity; (**G**) same as (**F**) but performed in the presence of MPO inhibitor 4-ABH, bar = 50 µm; H- anti-MPO antibody staining of a thin section. Bar = 200 µm Magnification – 20x (**F**,**G**), 10x (**H**).

A total of two ruptured and six unruptured hBA were further subjected to imaging with µMR after obtaining 50–80 µm sections (Figs 7 and 8). An ultrashort TE (UTE) pulse sequence was chosen to decrease the T2* effects at 7T that interfered with Gd-mediated longitudinal water relaxation time shortening (Fig. 7A). In a relatively large hBA (Fig. 7).

we observed two major areas of MR signal enhancement, which correlated with the presence of adventitial angiogenesis (Fig. 7, asterisk) and luminal micro haemorrhage (Fig. 7, arrows), both of which contained MPO activity as confirmed by using a fluorescent substrate of MPO (Fig. 7C). Overall, the same trend was observed in the samples of ruptured as well as unruptured hBA. In general, fluorescent substrate localized in tissue areas containing MPO activity which matched very well with the areas of elevated MR signal determined by applying UTE pulse sequence or T1w-SE pulse sequences (Figs 7 and 8). Due to the extensive infiltration of neutrophils and NETs in the luminal clots, the MPO activity was easily detectable in the samples of ruptured hBA (Fig. 8). For example, the matrix of the clot appeared bright on T1w images (Fig. 8A) after treating with paramagnetic MPO substrate-sensor. The same area showed MPO activity resulting in red fluorescence after incubating with 5HT-Cy3, and the level of fluorescence decreased dramatically after adding the irreversible inhibitor of MPO (Fig. 8G).

## Discussion

In this study, we used a µMRI histology setup for determining the distribution of MPO, a molecular marker of active inflammation within the microarchitecture of very small surgically excised fragments of hBA. Molecular imaging of this essential enzyme’s catalytic activity has been made possible by linking of an MR detectable paramagnetic metal (chelated Gd(III) to a substrate of the MPO enzyme (5HT). Upon diffusion into the tissue, the Gd(III)-conjugated 5HT substrate is oxidized by MPO, and is then retained at the site primarily as a result of binding to proteins. As a result, the areas with higher water relaxation rates are generated within the areas of high MPO activity. Two versions of chelated Gd(III) conjugated to 5HT were tested in this study: an acyclic DTPA chelate (Gd-bis-5HT-DTPA) and a macrocyclic DOTA chelate (Gd-5HT-DOTAGA). The distinction between linear and macrocyclic Gd(III) chelates is clinically significant owing to the higher safety profile of macrocyclic formulations^23,24^. Gd-5HT-DOTAGA demonstrated a higher MPO-mediated molar longitudinal relaxivity (r1) increase due to a lower initial relaxivity of non-reacted Gd-5HT-DOTAGA at 0.47–1.4T. The differences between the relaxivities of non-modified versus MPO-oxidized Gd-5HT-DOTAGA substrate-sensor are what give rise to the changes in MR signal intensity between pre- and post-contrast µMRI images and thus the potential clinical utility of this MPO sensor.

We anticipated that testing of MPO substrate-sensors at clinically relevant (1.5 and 3T) field strengths would eventually be needed to obtain a clearer picture of how compounds will perform in a more standard clinical setting. The higher field strength of 7T has some advantages for MR imaging, i.e. the overall higher tissue signal-to-noise ratio essential for the imaging of extremely small histology sections. High-field MRI also presents obstacles such as the increase in the ratio of r2/r1 compared to lower field strengths, which results in lower post-contrast signal enhancement for a given dose of Gd(III)-based MRI contrast agent. Those limitations can be partially overcome with optimization of pulse sequences such an ultrashort echo time (UTE) sequence, which maximizes signal from tissues with exceedingly short transverse relaxation times. Other factors must also be considered in interpreting µMR imaging results acquired in this study. Since the thickness of sections intended for µMRI exceeded standard histology section thickness by a factor of 10, the registration of the two image sets is not always 1:1 and this imposes limitations on the interpretation of signal overlap between the two visualization modalities. However, this does not prevent registering of µMR images with fluorescence images of MPO activity distribution in the tissue (Fig. 7). Results presented here and obtained in hBA imaging using MPO substrate-sensors confirmed previous results showing positive correlation between normalized MPO enzymatic activity localized in aneurysmal microstructure, and the presence of neutrophil infiltration as determined by immunohistochemistry and vascular wall inflammation/hBA instability^21,25,26^. The presence of MPO activity in the blood of patients, mainly as a component of neutrophil NETs^27^ brought about by the cells of innate immune system is a known indicator of downstream acute vascular events including strokes^28^ and the increased risk of aneurysm rupture within a 5-year period^21^.

Cerebral aneurysms are characterized histologically by chronic inflammation and degenerative changes to the vessel wall which may contribute to aneurysm pathogenesis and rupture^29–32^. Numerous inflammatory cytokines such as interleukins^33,34^, monocyte chemoattractant protein 1 (MCP1)^35,36^, as well as activation of NF-kB^30,37^ have been shown mechanistically to contribute to the pathogenesis of cerebral aneurysms. The picture emerging from results presented here, as well as numerous studies obtained in animal models and humans, shows the pathology of aneurysm development and rupture is complex and that multiple signalling cascades occurring in a range of vascular cell types and the extracellular matrix are involved (reviewed in^38^). Vascular wall remodelling may occur early in response to chronic vascular wall stress resulting from haemodynamic abnormalities. Ensuing endothelial damage may trigger the up-regulation of transcription factor NF-kB in smooth muscle cells and recruitment of inflammatory cells releasing IL-1β, IL-8, TNFα and other pro-inflammatory mediators^39,40^. We confirmed the presence of many IL-8-positive cells that infiltrate hBA wall potentially in response to downstream signalling associated with NF-kB activation (Fig. 2). Interleukin-8 (CXCL8) is a potent neutrophil chemotactic factor^41^ additionally promoting angiogenesis suggesting active participation of neutrophils in aneurysmal wall remodelling^42^. The demonstrated use of µMRI for the detection of MPO activity *in situ* in non-fixed human tissue sections has applicability to the ever-increasing number of investigations that seek to understand the role of molecular and nanoparticle-assisted imaging in detecting the development and progression of brain vascular pathology^22,43^. The potential of µMRI as a preclinical and translational imaging modality is promising but is still in the early stages of being fully validated. However, we anticipate that high-resolution µMRI has a potential of becoming widely applicable in the development of new tissue-specific contrast agents and sensors involving direct interaction with the components of surgically excised human tissue samples. We foresee broad application of this technique in research directed at testing specific activity of novel MR enzyme-specific substrates in realistic *in vitro* model systems.

## Methods

This prospective study involving human pathology samples was approved by the University of Massachusetts Medical School (UMMS) Institutional Review Board (IRB) and patient’s written informed consent was required before surgery. HIPAA compliance was enforced throughout the study. The following inclusion criteria were applied: included were adult female and male patients (ages 18–79 years old) with symptomatic or asymptomatic, ruptured or unruptured saccular cerebral artery aneurysms for which microsurgical clipping has been deemed the most appropriate course of treatment. The following exclusion criteria were applied: traumatic or mycotic aneurysms, aneurysms in patients with concurrent adult polycystic kidney disease, aneurysms in patients with concurrent degenerative connective tissue disorders including cystic medial necrosis, fibromuscular dysplasia, Marfan syndrome, Turner’s syndrome, and giant cell and Takayasu’s arteritis, patients within a vulnerable population, including foetuses, neonates, pregnant women, patients unable to provide informed consent, institutionalized individuals and prisoners. A total of n = 10 surgically resected samples of human saccular aneurysms were included in the study (n = 7 female, n = 3 male, 2 ruptured, 8 unruptured, median age - 57 years; see Table 1). One frozen sample of AVM (sectioned) was generously provided by Drs. William L. Young and Hua Su (William L. Young, M.D., Department of Anesthesia and Perioperative Care, University of California, San Francisco).

**Table 1:** Characteristics of surgically obtained aneurysmal dome samples.

Patient number	Age	Sex	Location^(a)^	UR/R	Largest diameter, mm	µMRI	Immunohistochemistry markers		
							MPO	NETs^(b)^	calprotectin^(c)^
1	50	M	RMCA	R	11	+	+	ND	+
2	71	F	RMCA	R	11	+	+	ND	+
3	39	F	LMCA	UR	8	+	+	ND	−
4	71	F	RMCA	UR	6	+	+	+	+
5	62	F	LMCA	UR	9	−	+	−	+
6	76	M	LMCA	UR	6	−	+	+	+
7	51	F	ACOM	UR	3	−	+	+	−
8	52	F	LMCA	UR	7	+	+	+	+
9	52	F	RMCA	UR	4	+	+	+	+
10	74	M	ACOM	UR	8	−	−	+	+

^(a)^RMCA- Right middle cerebral artery; LMCA- left middle cerebral artery; ACOM - anterior communicating artery; UR- unruptured, R- ruptured.

^(b)^Neutrophil extracellular traps (NETs) were detected by using digoxigenin-labeled anti-histone H3 (citrulline R2 + R8 + R17) antibody, (ab5103 Abcam).

^(c)^S100 calcium-binding proteins (expressed by granulocytes, monocytes and macrophages) were detected by using anti-calprotectin (S100A8/A9 complex) antibody (ab22506, Abcam); ND- not done.

### Myeloperoxidase substrate synthesis and testing

Fluorescent substrate 5HT-Cy3, (5-hydroxytryptamide of Cy3) was synthesized by using a method described in ^44^. Briefly, 5HT-Cy3 was synthesized by reacting 1.1 molar excess of 5-hydroxytryptamine with mono N-hydroxysuccinimide ester of Cy3 (GE Healthcare Bios-Sciences Corp., Piscataway NJ) in DMF in the presence of triethylamine under argon. The reaction product was precipitated using 10 volumes of acetone:diethyl ether (1:2 by vol), dried under argon and purified by using C18 HPLC (Discovery C18 column, Sigma, St. Louis MO) and a gradient of acetonitrile in 0.05 M triethylammonium acetate, pH 7.0.

Sodium salt of Gd-5HT-DOTAGA (gadolinium(III)-mono-5-hydroxytryptamide of 2,2′,2′′-(10-(2,6-dioxotetrahydro-2H-pyran-3-yl)-1,4,7,10-tetraazacyclododecane-1,4,7-triyl)triacetic acid) was synthesized in three steps: (1) the coupling of tert-butyl protected DOTAGA^45^ with serotonin hydrochloride by using the HATU coupling reagent and DIPEA as a base; (2) acidic deprotection with formic acid and hydrochloric acid of the protecting groups; (3) complex formation with Gd(III) by using gadolinium chloride in a sodium acetate buffer at pH 6. The purification was performed by using fast liquid chromatography on C18-reversed phase (ISCO CombiFlash) by using a gradient of acetonitrile in 0.1% formic acid with subsequent characterization by mass-spectrometry, (Agilent LCMS 6130) and gadolinium (III) content by inductively coupled plasma-mass spectrometry (Agilent ICP-QQQ 8800).

Gadolinium (III) DTPA bis-5-hydroxytryptamide (Gd-bis-5HT-DTPA) was synthesized and characterized as described in^12,46^. All substrates were purified using C18-HPLC and lyophilized prior to use. Test reactions in the presence of 6.6 U MPO (1100 U/mg, Meridian Life Sci, Memphis TN) and 1.2 U glucose oxidase (Millipore-Sigma, St. Louis MO) and various concentrations of MPO substrates were run for 3–18 h and subjected to analysis using relaxometry at 0.47T and 1.41T (Minispec mq20 and mq60, Bruker BioSpin Corp. Billerica MA) and gel electrophoresis as described in^13,47^.

### Histology

hBA and AVM surgical samples were obtained immediately post resection and were immersed in sterile saline. All hBA samples used in the study (n = 10) are listed in Table 1. Thin frozen sections (6–8 µm thick) were fixed in acetone (−20 °C). After washing in 50 mM Tris, 100 mM NaCl (TBS) aneurysm and AVM sample sections were incubated for 90 min in TBS containing 1 mM EDTA, pH 7 at 60 °C to inhibit endogenous phosphatase activity and blocked in 5% bovine serum in TBS, pH 7.5 for 2 h. Anti-human MPO mouse monoclonal antibodies (2C7), anti-calprotectin (S100A8/A9 complex) antibody (MAC387), Polyclonal rabbit anti-p65 NF-kB antibody (ab86299), anti- IL-6 mAb (ab9324), anti-IL-8 mAb (ab18672) all from Abcam, Cambridge MA, were incubated with the sections overnight at 1:200–1:300 dilutions in 5% bovine serum in TBS. The secondary antibody (anti-mouse rabbit- alkaline phosphatase conjugate, Roche) was used at 1:500 dilution. For identification of neutrophil extracellular traps (NETs) rabbit polyconal anti-histone H3 antibody (citrulline R2 + R8 + R17, ab5103 Abcam) was covalently modified by using N-hydroxysuccinimide ester of digoxigenin carboxylic acid (Roche Diagnostics, Indianapolis, IN) and purified on Bio-Spin P30 spin columns (Bio-Rad, Hercules CA) as described in^48^. Anti-digoxigenin F(ab′)_2_ alkaline phosphatase conjugate (Roche) at dilution 1:400 was used to identify digoxigenin-labeled primary antibodies. The staining was performed using a nitro-blue tetrazolium and 5-bromo-4-chloro-3′-indolyphosphate (NBT/BCIP) solution (Roche Applied Science) and counter-stained with nuclear fast red. Serial thick (80–100 µm) non-consecutive sections were subjected to µMRI imaging following incubation with 0.5 mM Gd-5HT-DOTAGA and 1 mM hydrogen peroxide for 30 min. Fluorescent staining of MPO-positive cells and staining of extracellular MPO in thin sections was performed by incubating the sections in the presence of 0.1 mM 5HT-Cy3/1 mM H_2_O_2_ with DAPI counterstaining of the nuclei. In control experiments specific irreversible inhibitor of MPO 4-aminobenzoic acid hydrazide was added to the substrate solution at 1 mM.

### MR microscopy

Thick sections of human brain vascular pathology specimens (Table 1, 80–100 µm thick) were treated as above in the presence of MPO imaging substrates (0.5 mM in PBS) for 30 minutes in the absence or in the presence of MPO inhibitors before being imaged with µMRI using a T1w-SE sequence. Direct µMRI of tissue sections was performed using a set of homebuilt histological coils tuned to operate at the Larmor frequency corresponding to 300 MHz and interfaced to a 200 mm horizontal bore 7 Tesla Bruker µMRI system (Bruker BioSpin) equipped with an actively shielded gradient coil insert 750-mT/m gradient strength, 100-μs rise time^9^. Depending on the sample (40-µm to 100-µm) and the histology coil used, the MRI in-plane spatial resolution ranged from 57-µm to 100-µm acquired within the overnight scan. Pulse sequences used: 2D T1w-GE with TE/TR 3.2/100 ms; 2D multi-echo T2*w-GE TE/ES/TR 4.9/4.2/100 ms.

### Statistics

Statistical analysis was performed by using Prism 7 (GraphPad Software Inc.) Results are expressed as mean ± SD. The analysis of means and assignment of statistical significance was be performed by using two-tailed unpaired t-test with Welch’s correction.

### Data availability

All data generated or analysed during this study are included in this published article (and its Supplementary Information files).

## Electronic supplementary material

Supplementary information
