# Supplementary material for: High-resolution Imaging of Myeloperoxidase Activity Sensors in Human Cerebrovascular Disease
Source: Sci Rep. 2018 May 16;8:7687. doi: 10.1038/s41598-018-25804-y (PMC5956082; doi:10.1038/s41598-018-25804-y)
Supplement: Supplementary file 1 — Supplementary information [file 41598_2018_25804_MOESM1_ESM.pdf]

Supplementary Information

# High-resolution Imaging of Myeloperoxidase Activity Sensors in Human Cerebrovascular Disease.

Youssef Z. Wadghiri[1], Minh Dung Hoang[1], Anita Leporati[2], Matthew J. Gounis[2], Aurora Rodríguez-Rodríguez[3], Mary L. Mazzanti[2], John P. Weaver[4], Ajay K. Wakhloo[2], Peter Caravan[3], Alexei A. Bogdanov,Jr[2]*.

[1]New York University School of Medicine, Center for Biomedical Imaging, New York, NY; [2]Department of Radiology, University of Massachusetts Medical School, Worcester MA; [3]A. Martinos' Center for Biomedical Imaging, Massachusetts General Hospital, Charlestown MA; [4]Department of Neurosurgery, University of Massachusetts Medical School, Worcester MA

*Author to whom correspondence should be addressed:

S6-434, Department of Radiology, University of Massachusetts Medical School

55 Lake Ave North, Worcester MA 01655

Tel. 508-856-5571

FAX 508-856-1860

Alexei.Bogdanov@umassmed.edu

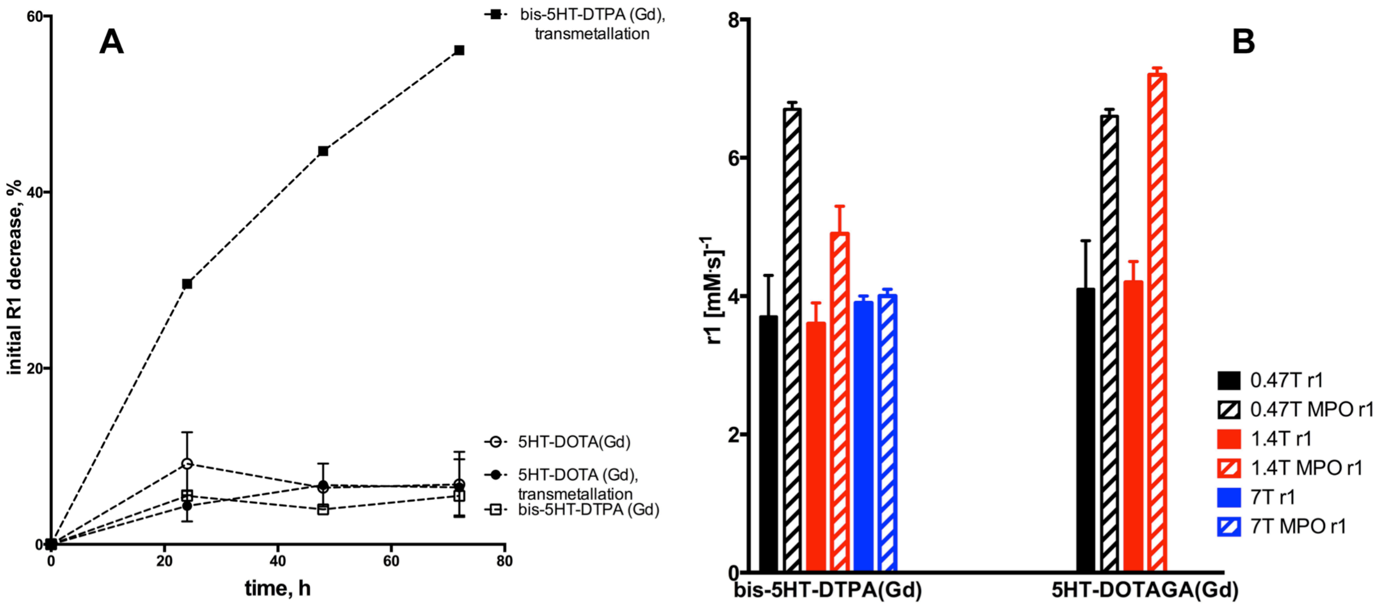


**Figure S1. Properties of paramagnetic MPO substrates.** (**A**) The decrease of R1 (longitudinal relaxation rate) in 0.25 mM solutions Gd-bis-5HT-DTPA and Gd-5HT-DOTAGA in the presence of the excess of Zn(II) and phosphate as a function of time. The increase of R1 in the case of Gd-bis-5HT-DTPAGA indicates gradual transmetallation on Gd(III). (**B**) Relaxivity of Gd-bis-5HT-DTPA and Gd-5HT-DOTAGA solutions (concentration range – 0.05-0.25 mM) before and after the addition of MPO and hydrogen peroxide measured at various magnetic field strengths (Bo). The products of MPO reaction (hatched bars) show higher molar longitudinal relaxivity (r1) than the initial products at 0.47 and 1.41T but not at high-strength magnetic field (7T). Results are shown as mean±SD (n=3).
